# Supplementary figures and images for: Effect of thermal control of dry fomites on regulating the survival of human pathogenic bacteria responsible for nosocomial infections
Source: PLoS One. 2019 Dec 27;14(12):e0226952. doi: 10.1371/journal.pone.0226952 (PMC6934310; doi:10.1371/journal.pone.0226952)

Supporting information: S1 Fig

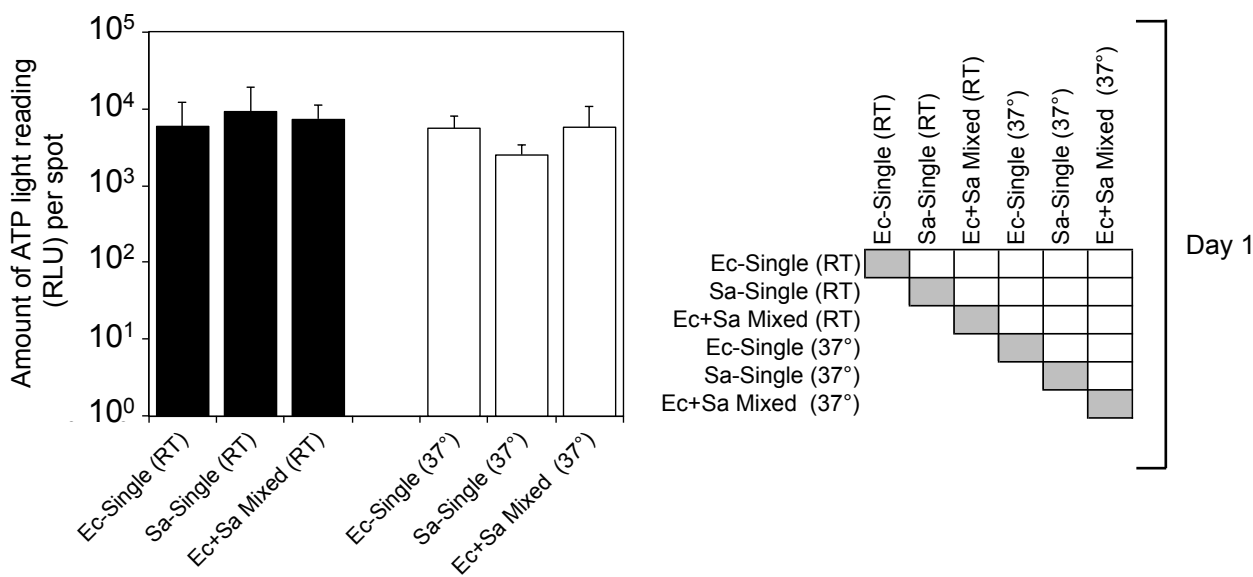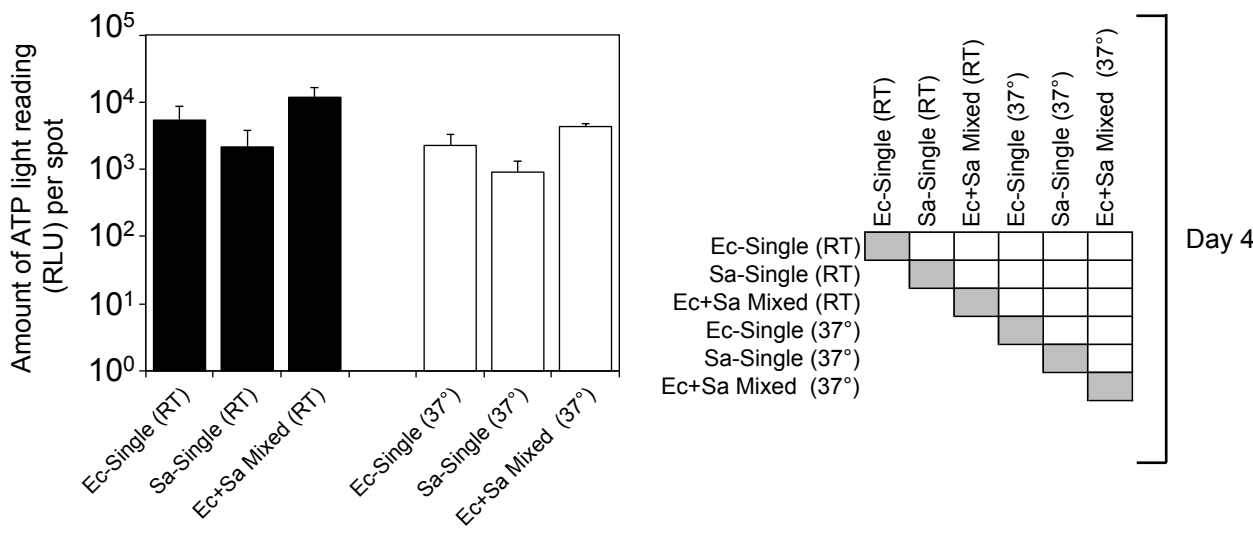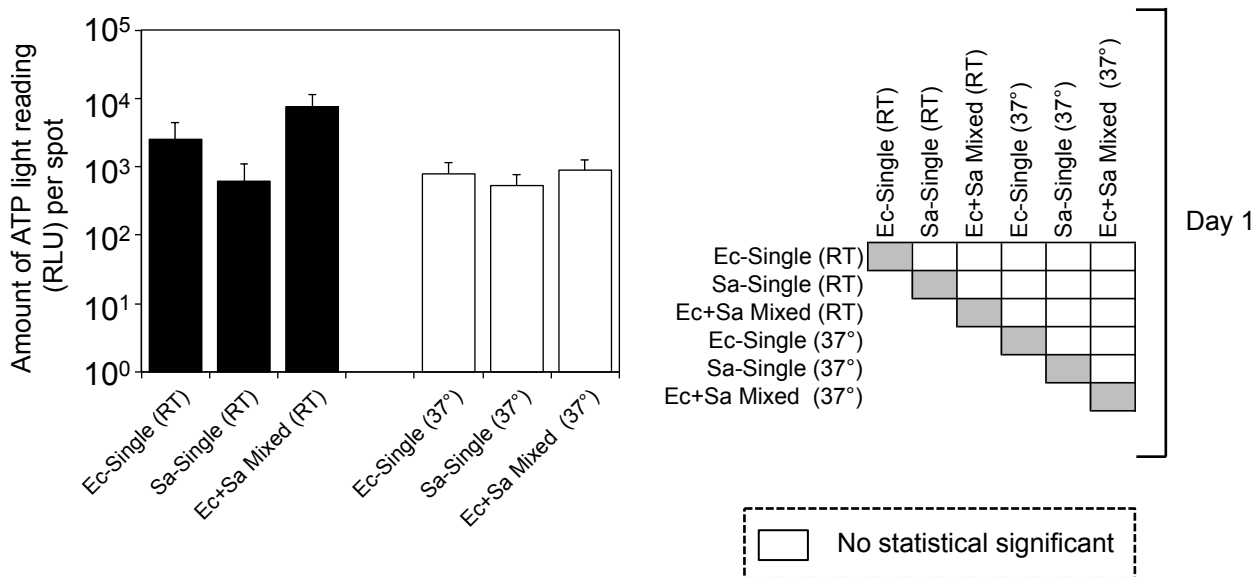

Supplement: S1 Fig — The amount of ATP was determined using a Clean-Trace Luminometer (3M, USA), and the values were expressed as relative light units (RLUs), according to a previously described protocol [17]. Ec, E. coli ATCC 25922. Sa, S. aureus ATCC 29213. RT, room temperature. (PDF) [file pone.0226952.s001.pdf]

Supporting information: S2 Fig

A

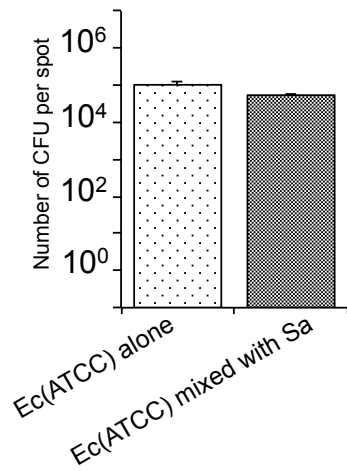

B

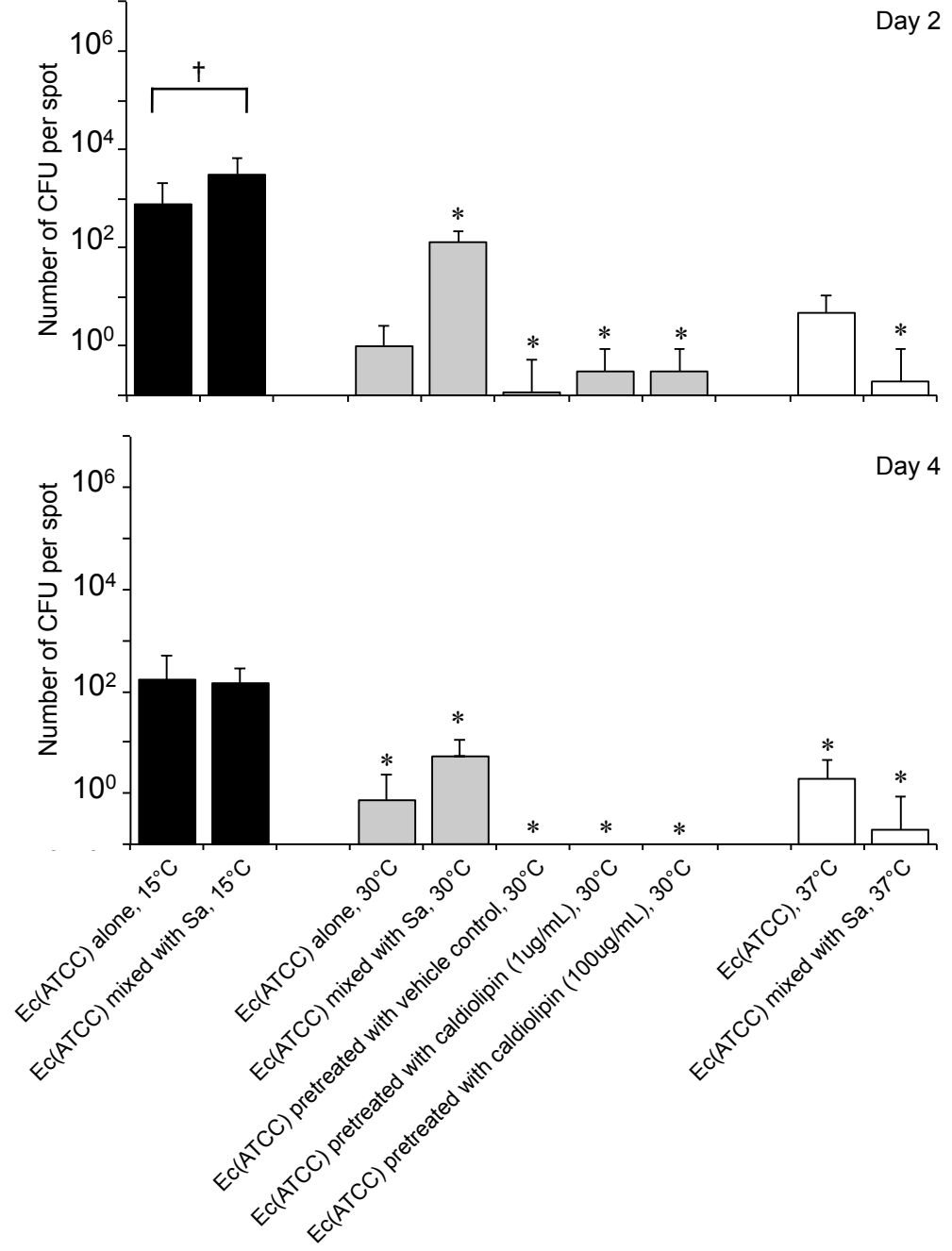

Supplement: S2 Fig — Black and dashed bars show the CFU numbers of E. coli and S. aureus, respectively. A. Bacterial numbers immediately (within 20 min) after drying. Ec, E. coli (ATCC 25922). Sa, S. aureus (ATCC 29213). B. Changes in bacterial numbers at distinct temperatures (15°C, 30°C, 37°C) over 11 days incubation, as analyzed by a CFU assay. Ec, E. coli (ATCC 25922). Multiple comparisons of the data were assessed by Bonferroni/Dunn analysis. Asterisk indicates a statistically significant difference compared with the value at 15°C. *, p<0.05. There was no statistically significant difference between the values at 30°C, regardless of the presence of cardiolipin. †, p<0.05. Ec, E. coli ATCC 25922. Sa, S. aureus ATCC 29213. (PDF) [file pone.0226952.s002.pdf]

Supporting information: S3 Fig

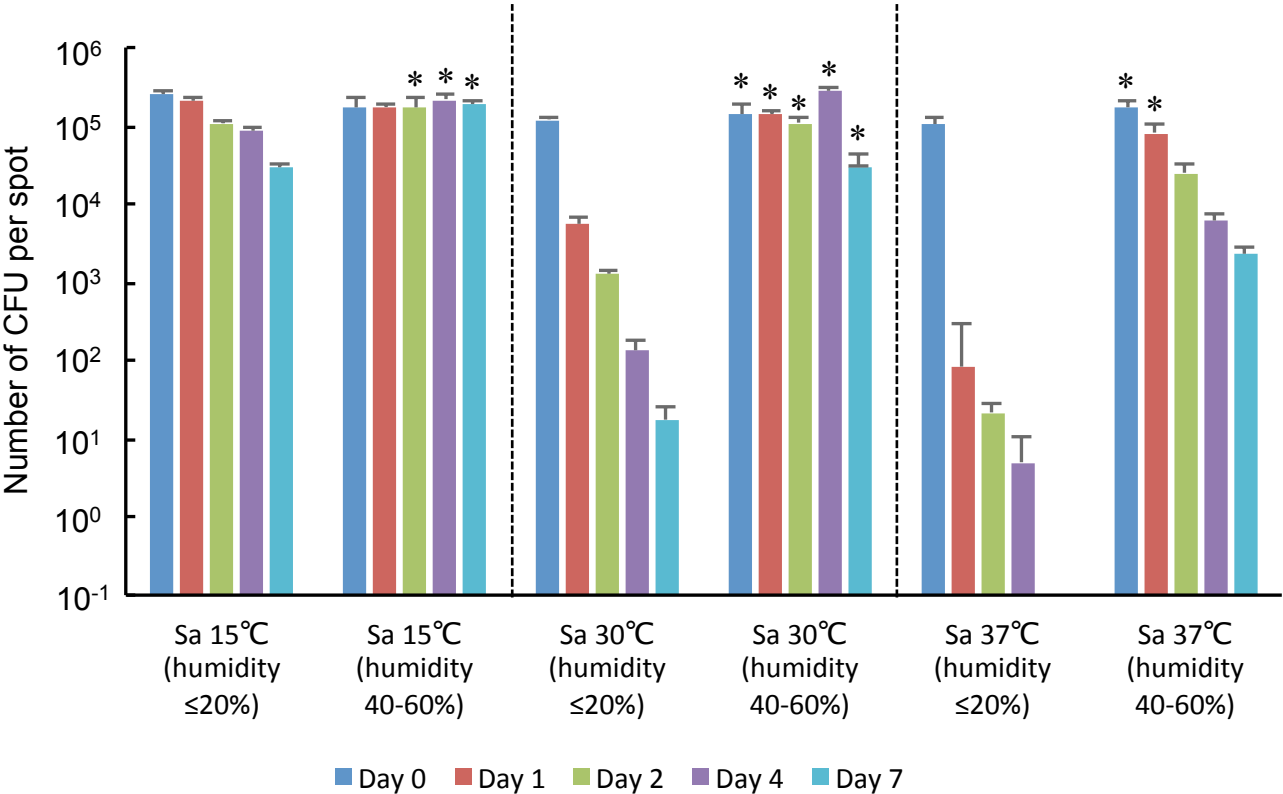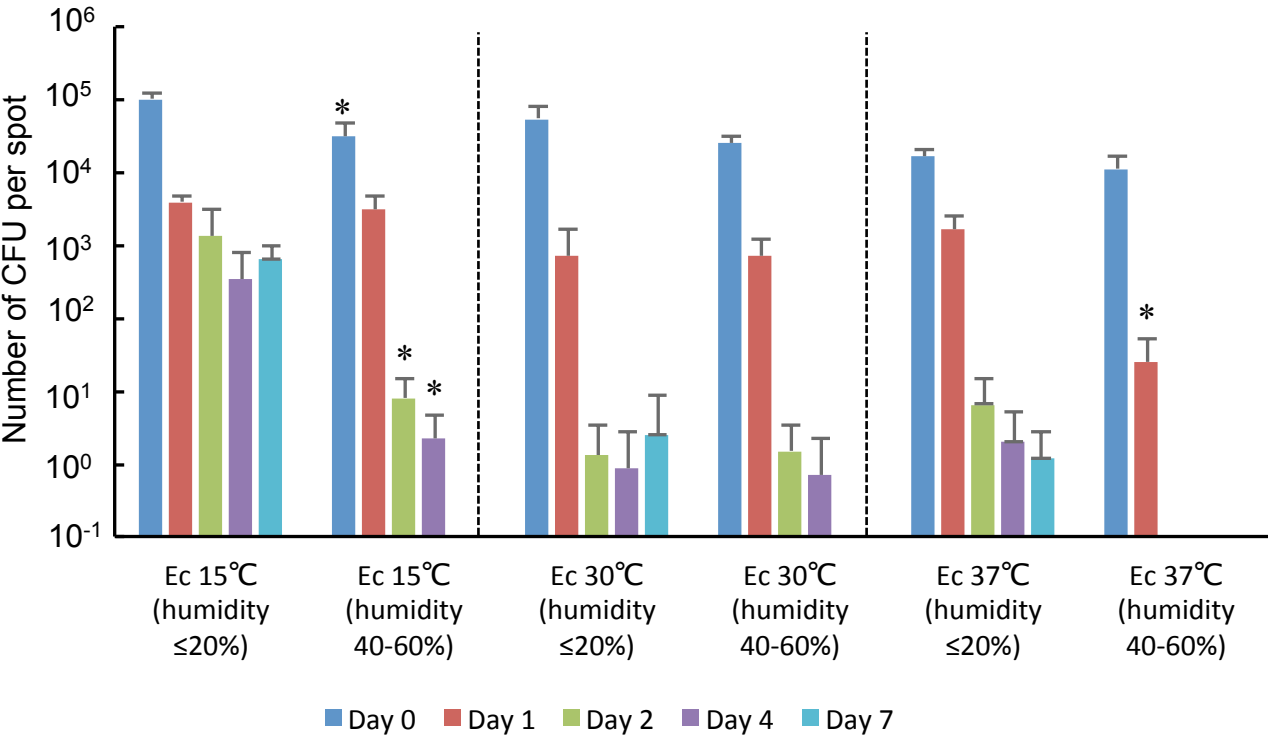

Supplement: S3 Fig — Changes in bacterial numbers at distinct temperatures (15°C, 30°C, 37°C) with or without humidity control over 7 days, as determined by a CFU assay. Upper panel. Ec, E. coli (ATCC 25922). Lower panel. Sa, S. aureus (ATCC 29213). *, p<0.05 vs. the values without humidity control. See the Materials and methods. (PDF) [file pone.0226952.s003.pdf]

Supporting information: S4 Fig

A

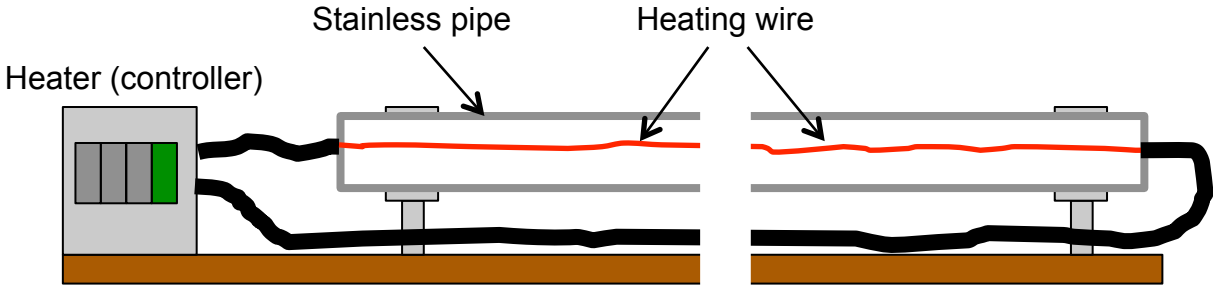

B

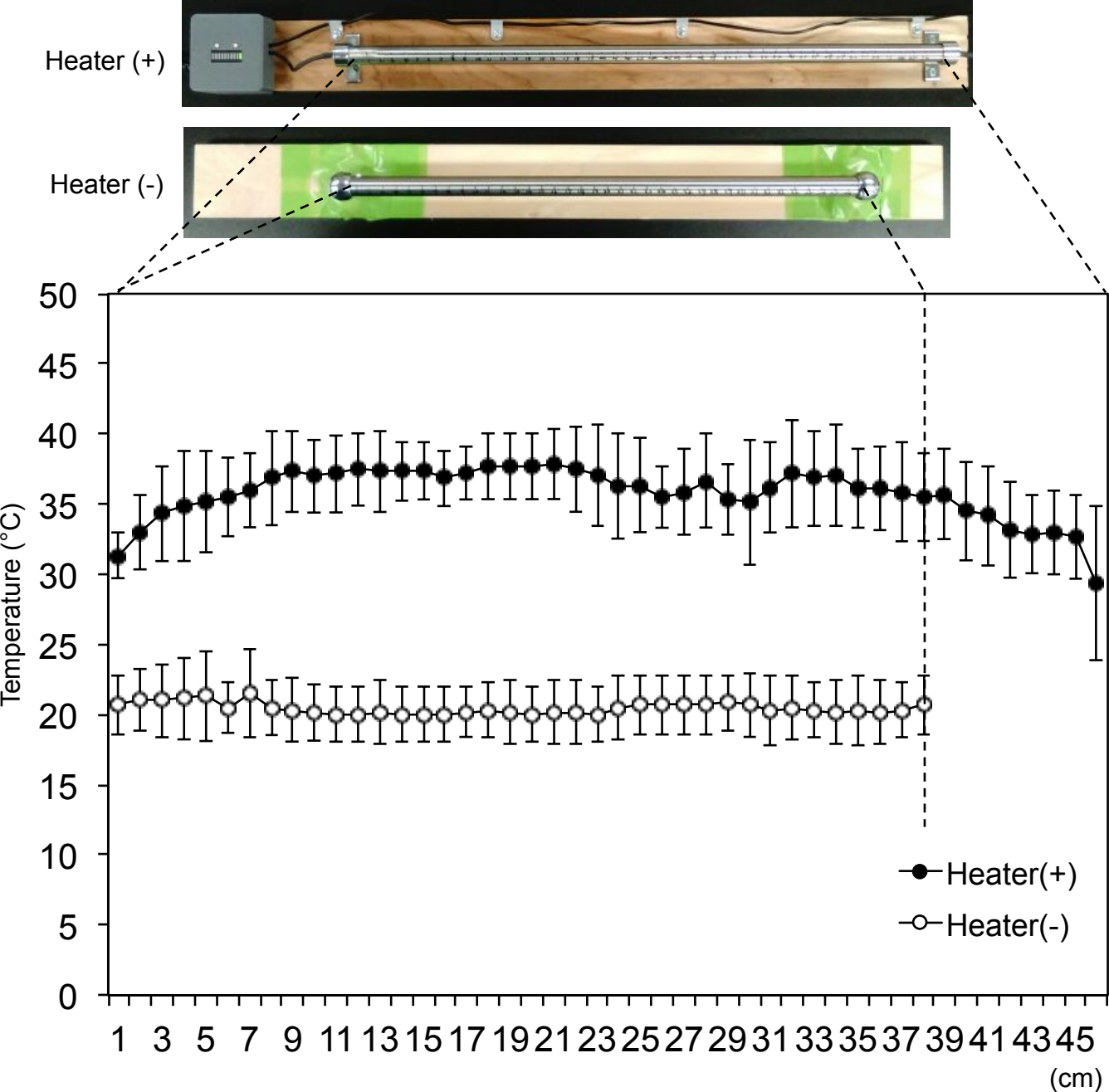

Supplement: S4 Fig — A. Structure of the handrail pipe with the heater. The stainless steel handrail pipe (diameter, 13 mm; thickness, 2 mm) was equipped with a heater wire at the center of the pipe to control the dry surface at body core temperature. B. Comparison of the surface temperature of the handrails with and without heaters. Temperatures were monitored by a hand-held infrared sensor (CT-2000D). Each of the values is shown as an average with the standard deviation. Black and white circles are shown as the values with and without heaters, respectively. (PDF) [file pone.0226952.s004.pdf]

Supporting information: S5 Fig

Heater (-)

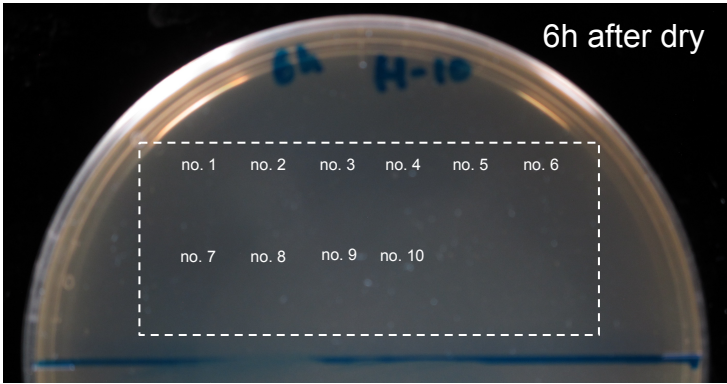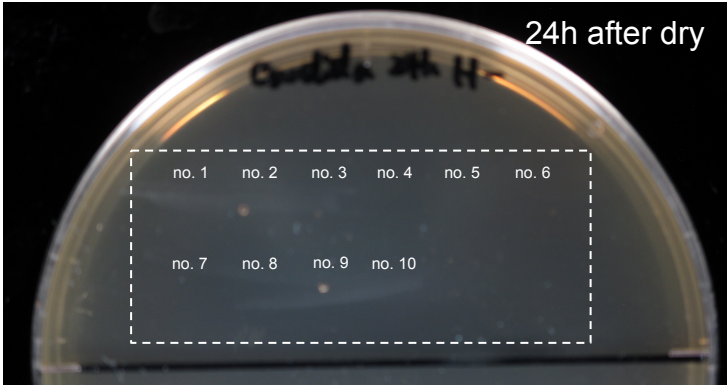

Heater (+)

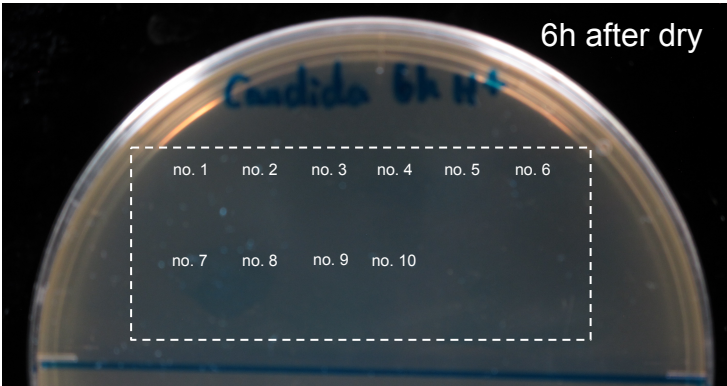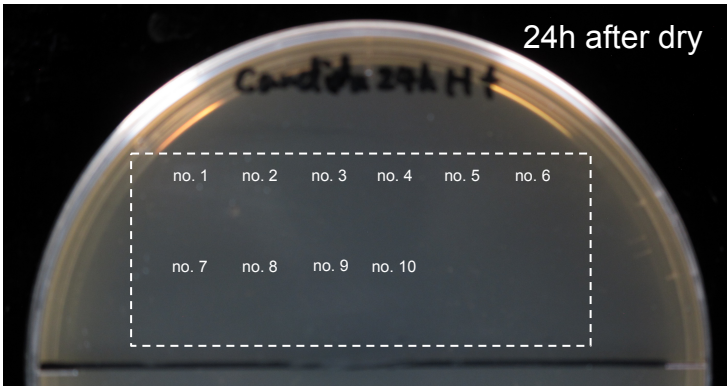

Supplement: S5 Fig — Images show the effect of thermal control on the survival of C. albicans on the handrail device 6 and 24 h after drying with [Heater (+)] or without thermal control [Heater (˗)]. Numbers (no. 1–10) show the yeast-like fungus spots on the PDA agar. (PDF) [file pone.0226952.s005.pdf]
